# The GCN4-Swi6B module mediates low nitrogen-induced cell wall remodeling in *Ganoderma lucidum*

**DOI:** 10.1128/aem.00164-25

**Published:** 2025-03-27

**Authors:** Lingyan Shi, Lingshuai Wang, Rui Liu, Jing Zhu, Liang Shi, Ang Ren, Huhui Chen, Mingwen Zhao

**Affiliations:** 1 Key Laboratory of Agricultural and Environmental Microbiology, Ministry of Agriculture and Rural Affairs, College of Life Sciences, Nanjing Agricultural University 98430, Nanjing, China; University of Illinois Urbana-Champaign, Urbana, Illinois, USA

**Keywords:** GCN4, Swi6, cell wall thickness, low nitrogen, *Ganoderma lucidum*

## Abstract

In natural habitats, microorganisms encounter various unfavorable environmental stresses, including nitrogen deficiency. As the outermost barrier, the cell wall plays a crucial role in the interaction between the cell and the external environment. However, the effect of low nitrogen on cell wall thickness, especially the underlying molecular mechanism, is unclear. Here, we found that compared with those under normal nitrogen conditions, both the cell wall thickness and polysaccharide content of *Ganoderma lucidum* are increased under low nitrogen conditions. Furthermore, the abundance of SWI6B, a transcription factor that participates in cell wall remodeling, is also increased in low-nitrogen environments. The thickness and polysaccharide content of the cell wall increased in *SWI6B*-overexpression strains (*SWI6B-OEs*) but decreased in *SWI6-*knockdown strains (*swi6-kds*). Moreover, although the cell wall thickness of all the genotypes increased under nitrogen-limited conditions, the percentage of upregulated *swi6-kds* was significantly lower than that of the WT, and the percentage of increased *SWI6B-OEs* was the highest. Moreover, GCN4, a key transcription factor of the low-nitrogen signaling pathway, was found to directly bind to the promoter of *SWI6*. The transcriptional and translational levels of *SWI6B* were reduced in *GCN4*-knockdown strains (*gcn4-kds*), indicating a positive regulation of *SWI6B* by GCN4. Consistently, the cell wall thickness of *gcn4-kds* was also lower than that of the wild type. Taken together, our results revealed that the GCN4-Swi6B module regulates cell wall remodeling in *G. lucidum* under nitrogen deficiency conditions.

**IMPORTANCE:**

To survive in stressful environments, fungi initiate cell wall remodeling pathways to adaptively modify the cell wall composition and structure. Here, we found that nitrogen deficiency upregulated the cell wall polysaccharide content and cell wall thickness through the GCN4-SWI6B signaling pathway. Our findings provide valuable insights into the environmental adaptation of fungal cell walls, contributing to a deeper understanding of fungal responses to environmental stress.

## INTRODUCTION

The cell wall is the outermost barrier of plant and microbial cells and can relieve external environmental pressure and maintain the shape of the cell through dynamic remodeling (1–3). The cell wall also controls material exchange to maintain intracellular and extracellular homeostasis (4, 5). Owing to mobility restrictions, microorganisms and plants often encounter various unfavorable environments throughout their lifespans, including drought (6–11), salt injury (12–14), and nutrient deficiency (15, 16). In response to adverse environments, the cell wall perceives external environmental factors and transmits signals, inducing cell wall remodeling to ensure the survival of organisms (17). For example, maize reshapes the cell wall to cope with salt stress by reducing the degree of feruloylation of arabinoxylan and altering the composition of lignin monomers (18). During yeast fermentation, ethanol exposure activates the cell wall integrity pathway, which further induces the upregulation of enzymes related to cell wall synthesis, such as β−1,3-D-glucan synthase FKS2, the chitin transglycosylase CRH1, and the O-glycosylated cell wall protein PIR3, leading to cell wall structural remodeling (19). In the cell wall integrity pathway, transcription factors play a key role in regulating gene expression. In *Magnaporthe oryzae*, the transcription factor Swi6 is necessary for cell wall integrity and pathogenicity (20). In *Aspergillus oligospora*, protein kinase C (PKC) interacts with and phosphorylates Swi6 to regulate its transcriptional activity, thereby improving cell wall integrity (21). In the edible fungus *Ganoderma lucidum*, the *SWI6* gene undergoes alternative splicing to form two different mRNA variants, *SWI6A* and *SWI6B*. Knockdown of *SWI6* via the RNA interface results in reduced cell wall thickness, but only *SWI6B* overexpression can increase cell wall thickness (22). Furthermore, compared with the WT strain, the overexpression of *SWI6B* reduced the sensitivity of *G. lucidum* to cell wall-disturbing agents, whereas *SWI6-*knockdown strains were more sensitive to these agents (22).

Nitrogen is a key nutrient required for the growth of fungi and plants and plays a crucial role in basal metabolism and tissue development. Nitrogen deficiency or deprivation is an unfavorable environment for fungi and plants and can directly regulate the cell wall structure. The average cell wall thicknesses of N-deprived microalgal *Nannochloropsis* sp. and *Chlorococcum* sp. were greater than those of N-replete cells (23). The cell wall thickness and components of plants are also increased by low nitrogen (16, 24, 25). Nitrogen deficiency is an important factor limiting fungal growth in natural habitats and has been reported to trigger morphological transitions in *Saccharomyces cerevisiae* (1, 4), *Aspergillus nidulans* (5), and *Neurospora crassa* (2). In *Cryptococcus neoformans*, nitrogen deficiency also increased the transcript levels of multiple genes related to the cell wall integrity pathway (26). These results indicate that nitrogen plays a crucial role in regulating cell wall composition and structural formation, but the underlying mechanism is still unclear.

To efficiently absorb and utilize nitrogen-containing nutrients in the environment, genes related to nitrogen absorption and metabolism are precisely regulated. Previous studies have shown that at least two nitrogen metabolism regulatory pathways are associated with transcription factors in fungi. The first pathway is the nitrogen catabolite repression pathway, which employs the transcription factor NIT-2/AreA as a key component (24, 25). AreA activates genes in the absence of priority nitrogen sources (such as glutamine, ammonium, glutamate, and asparagine), whereas NIT-2 responds to specific nitrogen sources and activates related genes (27). Therefore, the nitrogen catabolic repression pathway regulates fungal metabolism to utilize a wide range of nitrogen nutrients and promote fungal growth. The second pathway involves the general control nonrepressible-4 (GCN4), which can regulate amino acid biosynthesis and nitrogen utilization and is known as the general amino acid control pathway (28). Under low-nitrogen conditions caused by amino acid starvation, GCN4 activates the expression of more than 500 genes involved in nitrogen biosynthesis, the tricarboxylic acid cycle, and glycolysis pathways in yeast (29). When *Penicillium oxalicum* is grown in a nitrogen-deficient environment, the GCN4 homologous protein CpcA promotes lysine biosynthesis by inhibiting the synthesis of penicillin, thus improving intracellular nitrogen utilization (30). Previously, we reported that low nitrogen can activate *GCN4* transcription in *G. lucidum* (31). GlGCN4 interacts with SKO1 to form a transcriptional regulatory complex, which promotes the utilization of nitrogen under low-nitrogen conditions by increasing the transcriptional level of *AreA* (32). Moreover, GCN4 directly activates the expression of the mitochondrial pyruvate carrier, thereby regulating the tricarboxylic acid cycle and secondary metabolism of *G. lucidum* under nitrogen-limiting conditions (33). Some target genes regulated by GCN4 can also respond to both amino acid stress and oxidative stress. For example, GCN4 can directly bind to the promoter regions of the antioxidant genes GR, GST2, and CAT3 to increase their transcription levels, alleviating the accumulation of reactive oxygen species (ROS) under low-nitrogen conditions (31).

In this study, to investigate the cell wall changes under low-nitrogen conditions and understand the underlying molecular mechanisms, *G. lucidum* was cultured on solid media supplemented with different concentrations of asparagine (Asn) as a unique nitrogen source. The results demonstrated that the cell wall thickness under 3 mM Asn (low nitrogen) conditions was greater than that under 60 mM Asn (normal nitrogen) conditions. Furthermore, the expression levels of *GCN4* and *SWI6B* are also increased under low-nitrogen conditions. The GCN4 protein directly binds to the promoter and increases the transcriptional level of *SWI6B*. Reducing the expression levels of *GCN4* or *SWI6* via the RNA interface leads to a decrease in cell wall thickness. Together, our results reveal the molecular mechanism underlying the regulation of cell wall remodeling by the GCN4-SWI6B module under low-nitrogen conditions.

## MATERIALS AND METHODS

### Experimental strains and culture conditions

The wild-type (WT) *G. lucidum* strain was obtained from the Agricultural Culture Collection of China (Nanjing). The strains were cultured on complete yeast extract (CYM) medium (1% maltose, 2% glucose, 0.2% yeast extract, 0.2% tryptone, 0.05% MgSO_4_·7H_2_O, 0.46% K_2_HPO_4_, and 20% agar) at 28°C. For nitrogen treatment, the CYM medium was modified (2% glucose, 0.1% MgSO_4_·7H_2_O, 0.46% K_2_HPO_4_, 20% agar, and 3 mM or 60 mM asparagine as the sole nitrogen source).

For RNA and protein extraction, a two-stage cultivation strategy was used for fermentation experiments. First, mycelia were grown in liquid CYM in a flask with shaking for 4 days at 28°C. Second, the mycelia were washed with sterile ddH_2_O and then transferred to a modified liquid CYM medium containing 3  mM or 60  mM Asn as the sole nitrogen source in a flask with shaking for 5 days at 28°C. Mycelia were collected and used in the RNA and protein extraction experiments.

### Generation of transgenic strains

The gene overexpression vector pGPiE and the knockdown vector pMi were the same as in a previous report (32). To generate *GCN4*-overexpression strains, the *GCN4* CDS was amplified by PCR with the primers GCN4-OE-F/R (Table S1) and subsequently cloned and inserted into the pGPiE vector. To generate GCN4-knockdown strains, a specific genomic DNA fragment of *GCN4* was amplified by PCR with the primers GCN4-kd-F/R (Table S1) and cloned and inserted into the pMi vector. The GCN4 knockdown and overexpression vectors were subsequently transformed into *G. lucidum* using *Agrobacterium tumefaciens*-mediated transformation. Transgenic strains were selected by hygromycin resistance, and two independent knockdown or overexpression strains with the best efficiency were selected for subsequent experiments. Real-time quantitative PCR (RT-qPCR) and western blotting were used to verify the genetic strains. The genetic strains of SWI6 were generated using the same procedure. The empty vector pMi was used to generate the corresponding Sicontrol strain.

### Total RNA extraction and gene expression analysis

The mycelia of each strain were used to extract total RNA with the RNAiso Plus Reagent (TaKaRa). Total RNA was reverse transcribed with All-In-One RT MasterMix for cDNA synthesis (ABM) to generate cDNA. RT-qPCR was performed on a Mastercycler ep Realplex (Eppendorf) using cDNA and EvaGreen 2X qPCR MasterMix (ABM). The chitin and glucan synthase genes regulated by Swi6B were selected according to a previous study (32). The expression of these selected genes was analyzed via RT-qPCR. Gene expression was evaluated by the cycle threshold (Ct) value. The housekeeping gene 18S rRNA was used as an internal reference gene. To quantify the relative expression levels of the target genes, we used the 2^–∆∆Ct^ method according to a previous report (34). For the same sample, the Ct value of 18S rRNA was used to normalize the Ct values of the target genes. The value of the target gene in WT under normal nitrogen conditions was set to 1 for comparison with the values of this gene in different strains and conditions. The gene-specific primers used are listed in Table S1. The results are obtained from two biological replicates, each consisting of three technical replicates. The data are represented as the mean ± SD and were statistically analyzed as indicated in the figure legend.

### Quantification of cell wall β-1,3-D-glucan

The content of β−1,3-D-glucan was quantified according to previous reports (35, 36), with slight modifications. Mycelia from strains under different conditions were collected and washed with 0.1 M NaOH and lyophilized for 16 h. The lyophilized mycelia powder (0.01 g) was resuspended in 250 µL of 1 M NaOH and sonicated for 30 s, followed by incubation at 52 ° C for 30 min. Subsequently, 50 samples were mixed with 185 µL of aniline blue mixture (0.067% aniline blue, 0.35 N HCl, 0.98 M glycine-NaOH, pH 9.5) in a 96-well fluorescent plate at 52°C for another 30 min. The plates were cooled at 25°C for 30 min before fluorescence reading. The fluorescence values were recorded with a microporous plate-detecting instrument (Cytation 3, BioTek), with excitation and emission wavelengths of 405 nm and 460 nm, respectively. To normalize the values, the β−1,3-D-glucan analog curdlan was used to generate a standard curve. The values are expressed as the percent change in the relative fluorescence of mycelial tissue. The glucan content of the wild type under normal nitrogen conditions was set to 100%. The results are from three independent replicates (mean ± SD).

To obtain the increased percentage of β−1,3-D-glucan content, the values described above were used for calculation as follows: [(value^LN^ − value^NN^) / value^NN^]. Statistical significance was examined via one-way analysis of variance (ANOVA) and Tukey’s multiple comparison tests.

### Quantification of cell wall chitin

The determination of the chitin content was conducted according to previous reports (36), with slight modifications. Mycelia were harvested, washed, and lyophilized as described above. Ten milligrams of lyophilized mycelia were resuspended in 3 mL of saturated KOH and incubated at 130°C for 60 min. After cooling, 5 mL of precooled 75% ethanol was added and vortexed for 2 min, followed by incubation at 0°C for 15 min. The mixture was resuspended in 300 µL of 13.3% (wt/vol) Celtite 545 (68855-54-9, Sangon Biotech), and then centrifuged at 1,500 × *g* for 5 min at 4°C. The supernatant was discarded, and the pellet was washed once with 5 mL of 40% ethanol (ice-cold), twice with 10 mL of deionized water (ice-cold), and then centrifuged at 1,500 × *g* for 5 min at 4°C. The pellet was resuspended in 0.5 mL of deionized water and used in the subsequent steps.

Because chitin is equivalent to glucosamine, the glucosamine content was used to represent the chitin content. For every sample, a water-only sample and a sample with a known concentration of glucosamine (10 µg/mL) were used as standards. Standard samples consisting of water and glucosamine were used in all the following steps. After the pellet was resuspended in 0.5 mL of deionized water, 1 mL of extraction buffer (5% [wt/vol] NaNO_2_, 5% [wt/vol] KHSO_4_) was added, and the mixture was gently mixed on a rotator for 15 min. After centrifugation at 1,500 × *g* for 2 min at 4°C, 150 µL of the supernatant was collected and mixed with 450 µL of deionized water and 200 µL of 12.5% (wt/vol) NH_4_ sulfamate. The mixture was vortexed each minute for a 5 min period. After that, 0.2 mL of 3-methylbenzthiazolinone-2-hydrazone (5 mg/mL) was added to the mixture and incubated at 130°C for 3 min. The mixture was cooled at 25°C, 200 µL of 0.83% (wt/vol) ferric chloride was added, and the mixture was incubated at 25°C for 25 min before absorbance measurement. The absorbance at 650 nm was recorded with a microporous plate detecting instrument (Cytation 3, BioTek). The concentrations of glucosamine levels in the samples were calculated as follows: [(A650 sample − A650 water) × 10 µg/mL]/(A650 standard − A650 water). The results are from three independent replicates (mean ± SD).

To obtain the increased percentage of chitin content, the values described above were used for calculation as follows: [(value^LN^ − value^NN^) / value^NN^]. Statistical significance was examined using one-way ANOVA and Tukey’s multiple comparison tests.

### Microscopy assay

Mycelia cultured in a liquid medium containing 3 mM Asn or 60 mM Asn were collected and prepared as described previously for transmission electron microscopy (TEM) observation (37). Briefly, mycelia were collected and fixed with 2.5% glutaraldehyde in 0.1 M phosphate-buffered saline (PBS), pH 7.2. The fixed mycelial pellets were cut with an ultramicrotome and stained with 1% tannic acid (filtered aqueous solution), 2% uranyl acetate (filtered aqueous solution), and lead citrate in sequence. Sections of the cell wall were examined with H-7650 transmission electron microscopy (TEM) (Hitachi). The digital images were acquired using a Veleta camera and iTEM software, and the thickness was measured using ImageJ software (version 8.0). Three biological replicates of each strain were used for detection. Each biological replicate included at least 10 TEM images of individual cells.

### Total protein extraction and western blotting assay

Mycelia of each strain were used as samples for total protein extraction. Approximately 100 µg of mycelia was ground in liquid nitrogen and incubated with 800 µL of protein extraction buffer (PEB, 150 mM NaCl, 0.5% Tween-20, 1 mM EDTA, 1 mM DTT, 1 mM PMSF, 50 mM Tris-Cl, pH 7.5, and 1× Protease Inhibitor Cocktail) for 30 min in an ice bath. After two cycles of centrifugation (10 min, 13,000 rpm, 4°C), the supernatant was collected, and the protein concentration was determined via the Bradford dye reagent (Bio-Rad). Proteins were separated by 12% SDS-PAGE and transferred to polyvinylidene difluoride (PVDF) membranes. The membranes were blocked and incubated with specific antibodies as indicated. Images were acquired using a ChemiDoc Touch imaging system (Bio-Rad) and quantified using ImageJ software. The previously reported antibodies against α-GCN4 (32), *α-Actin* (38), *α-Swi6A,* and *α-Swi6B* (22) were used to detect the specific proteins.

### Yeast one-hybrid (Y1H) library screening and verification

Y1H library screening was performed with the Matchmaker Yeast One-Hybrid System (Clontech) according to previously described methods (39, 40) with minor modifications. The promoter region of the *SWI6* gene was analyzed with the JASPAR database (http://jaspar.genereg.net, accessed in 2021) to identify cis-elements and correlated transcription factors (41). The first 1,000 bp fragment (upstream of the start codon) containing several cis-elements was used in Y1H library screening. The fragment was subsequently subcloned and inserted into the pAbAi vector to generate the pAbAi-SWI6 vector (Invitrogen). Total RNA extracted from *G. lucidum* was used to construct a pGADT7-cDNA prey library. The pAbAi-SWI6 vector was transformed into Y1HGold yeast strains. After being grown in uracil-free SD media (SD/−Ura) for 3 days at 30°C, these Y1HGold yeast strains were used to generate competent cells, which were subsequently transformed with pGADT7-cDNA library vectors and subsequently grown on leucine-free SD media supplemented with 500 ng/mL aureobasidin A (AbA) (SD/−Leu/+AbA). Three days later, individual clones were separately collected and cultured in YPDA liquid media for plasmid extraction. The plasmids were used for DNA sequencing to obtain gene information. After the identification of *GCN4*, the CDS of *GCN4* was subcloned and inserted into the pGADT7-GCN4 vector and subsequently used in the Y1H assay to confirm the interaction between the GCN4 protein and the promoter of *SWI6*. All primers used in this experiment are listed in Table S1.

### Electrophoretic mobility shift assay (EMSA)

For the EMSA, DNA fragments of the promoter regions of *SWI6* were amplified with primers labeled with or without biotin probes (Shanghai Sangon Biotech). The open reading frame of the *GCN4* gene was subcloned and inserted into vector pET28a (Novagen) and expressed in the *Escherichia coli* strain Rosetta (DE3) to generate the recombinant GCN4-HIS protein. The recombinant protein was purified with Ni-charged MagBeads (GenScript) and used in the EMSA. The EMSA was performed with an EMSA kit (Thermo Scientific, 89880) according to the manufacturer’s instructions. All primers used in this experiment are listed in Table S1.

### Surface plasmon resonance (SPR) assay

SPR analysis was performed on a Biacore T200 system (GE Healthcare). GCN4 protein was diluted with running buffer (10 mM HEPES pH 7.4, 150 mM NaCl, 3 mM EDTA, 0.005% P20) at serial concentrations as indicated (7.81–125 nM). Diluted GCN4 was immobilized to theoretical resonance units (RUs) on S streptavidin-coated (SA) sensor chips (Cytiva, BR100531). The *SWI6* or m*-SWI6* probes were labeled with biotin and used as ligands at a concentration of 0.5 µg/µL. The binding kinetics were determined with a flow rate of 30 mL/min at 25°C. Full-curve dynamics fitting was performed with Biacore T2000 evaluation software to obtain the equilibrium dissociation constant (KD). The results were plotted in a sensor gram and expressed in RU against time.

### Chromatin immunoprecipitation qPCR (ChIP) analysis

Approximately 2 g of mycelia were cross-linked with 1% formaldehyde for 20 min and blocked with 125 mM glycine for 5 min, followed by washing with ice-cold PBS buffer for three cycles. The washed mycelial samples were ground to powder in liquid nitrogen and resuspended in ChIP lysis buffer (50 mM HEPES pH 7.5, 150 mM NaCl, 1 mM EDTA, 1% Triton X-100, 0.1% deoxycholate, 0.1% SDS, 1 mM PMSF, and 1× protease inhibitor cocktail) for 30 min at room temperature. The mixed solution was filtered through 40 µm nylon mesh and then centrifuged for 10 min (13,000 rpm, 4°C). The supernatant was collected and sonicated to obtain DNA fragments of approximately 100–500 bp. The sonicated DNA was diluted with ChIP dilution buffer (0.01% SDS, 1.1% Triton X-100, 1.2 mM EDTA, 16.7 mM Tris-HCl, pH 8.1, and 167 mM NaCl) and subjected to immunoprecipitation. Before immunoprecipitation, 1% of the solubilized chromatin was reserved as input DNA. The α-GCN4 antibody was coupled with protein G magnetic beads and then incubated in sonicated DNA solution for 6 h at 4°C. Moreover, to eliminate the nonspecific binding, an antibody corresponding to IgG was used as a control to conduct immunoprecipitation. After immunoprecipitation, the magnetic beads were separated with a magnet and subjected to proteinase K treatment for 2 h at 45°C. The immunoprecipitated DNA was extracted and quantified by qPCR. The data were analyzed using the comparative 2^−ΔΔCt^ method according to a previous report (42) and are presented as the fold enrichment over IgG. The experiment was independently performed three times. All primers used for ChIP‒qPCR are listed in Table S1.

### Statistical analysis

The experiment was repeated at least three times independently, except where indicated otherwise. The values are from three technical replicates (mean ± SD). Statistical analysis was performed with GraphPad Prism version 8.0 using one-way ANOVA and Tukey’s multiple comparison tests (different letters represent significant differences) or Student’s *t* tests (statistical significance is represented by asterisks) (*P* < 0.05).

## RESULTS

### Low nitrogen induced an increase in cell wall thickness and Swi6B abundance

**Fig 1:**
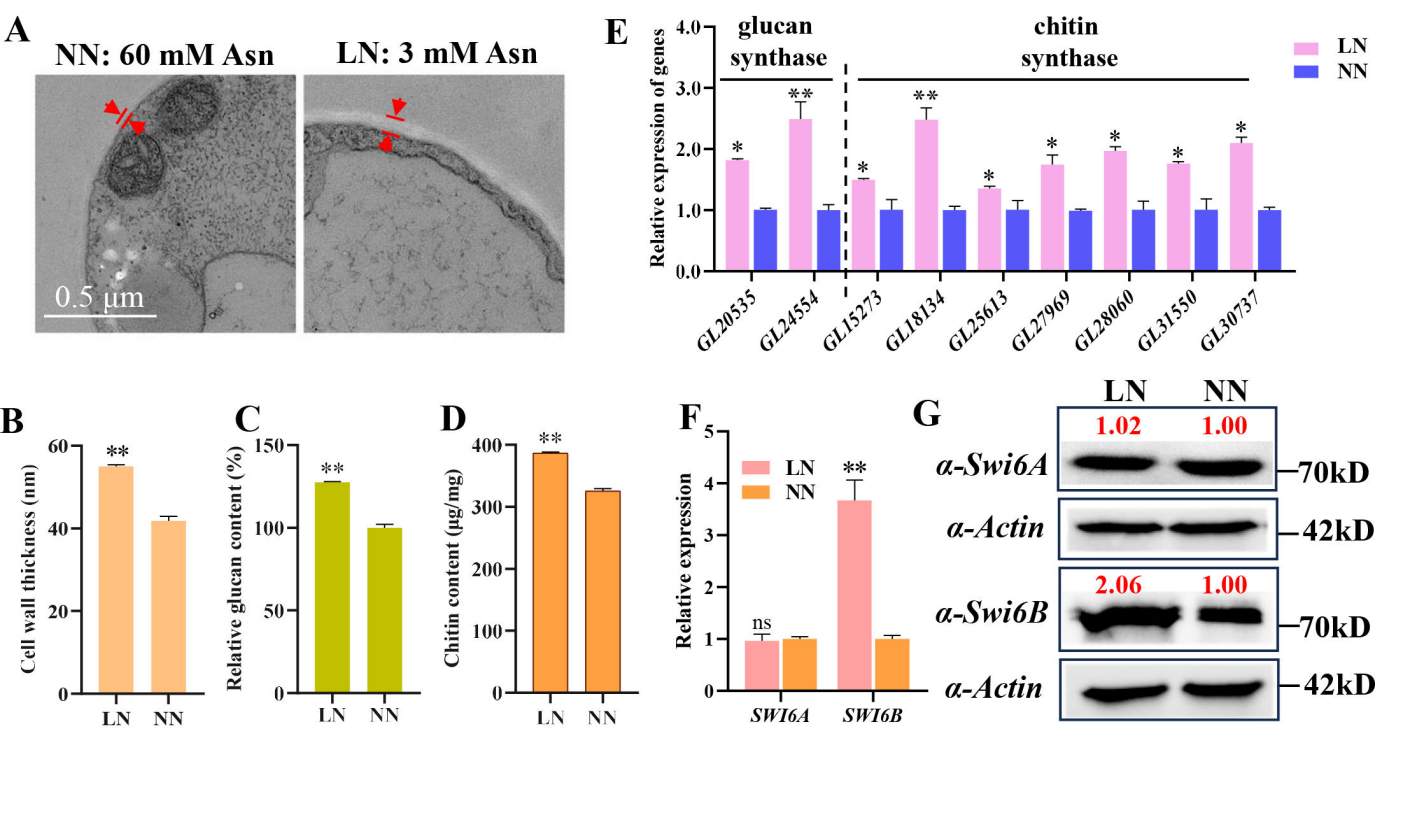
Low-nitrogen conditions upregulate the cell wall thickness (CWT), polysaccharide content, polysaccharide synthase genes transcription, and both the transcript and protein levels of Swi6B. Wild-type strain was grown on the CYM media supplemented with 3 or 60 mM asparagine (Asn). The mycelia were collected for RNA extraction and measurement of chitin and glucan content. (A, B) The representative pictures (A) and values (B) of CWT of WT. Values are shown as mean ± SD (*n* = 30). (C, D) The content of glucan (C) and chitin (D) of WT. (E) The relative transcription of genes coding for chitin synthases and glucan synthases. (F) RT-qPCR assays of the relative transcription level of the two *SWI6* splice isoforms in WT strain. (G) Western blotting analysis of the protein level of Swi6A and Swi6B in WT strain. The red number indicates the band intensity, and the protein level of WT under NN conditions was set as 1. For (A–G), LN, low nitrogen; NN, normal nitrogen. All the data were obtained from WT strain grown under LN or NN conditions as indicated. For (B–F), the values are shown as the mean ± SD (*n* = 3); the statistical significance is conducted with Student’s *t* test (*, *P*  <  0.05; **, *P*  <  0.01).

In natural habitats, the growth of *G. lucidum* is often regulated by nutrient deprivation. Low nitrogen has been shown to reduce the growth of *G. lucidum* (31). However, the effect of low nitrogen on the cell wall structure of *G. lucidum* is unclear. To explore this issue, we detected mycelial growth on modified CYM supplemented with Asn as the sole nitrogen source. For nitrogen treatment, 3 mM Asn was set as low nitrogen, and 60 mM Asn was set as normal nitrogen. The cell wall of the WT *G. lucidum* strain under different nitrogen concentrations was observed with TEM, and the cell wall thickness was further measured. The cell wall thickness under low-nitrogen conditions was significantly greater than that under normal-nitrogen conditions (Fig. 1A and B). The contents of both chitin and β−1,3-D-glucan, which are the main components of the fungal cell wall, were also quantified. The results demonstrated that both parameters increased under low-nitrogen conditions (Fig. 1C and D). Consistent with these findings, the transcription of the chitin synthase and glucan synthase genes was significantly induced by low-nitrogen conditions (Fig. 1E).

Previously, we reported that Swi6B plays an important role in promoting fungal growth and cell wall thickness under optimal growth conditions (22, 35). Swi6B regulates cell wall synthesis by promoting the transcription of the chitin and glucan synthase genes (22). Therefore, we further examined the effects of nitrogen starvation on *SWI6B* transcriptional and translational expression. In *G. lucidum*, the *SWI6* gene undergoes alternative splicing, resulting in two splice variants *SWI6A* and *SWI6B* (22). Our findings revealed that low-nitrogen conditions significantly increased *SWI6B* transcription and protein abundance but did not obviously affect SWI6A (Fig. 1F and G). These results suggest that Swi6B may play a crucial role in cell wall remodeling under low-nitrogen conditions.

### Swi6B improves cell wall thickness and polysaccharide accumulation under low nitrogen conditions

**Fig 2:**
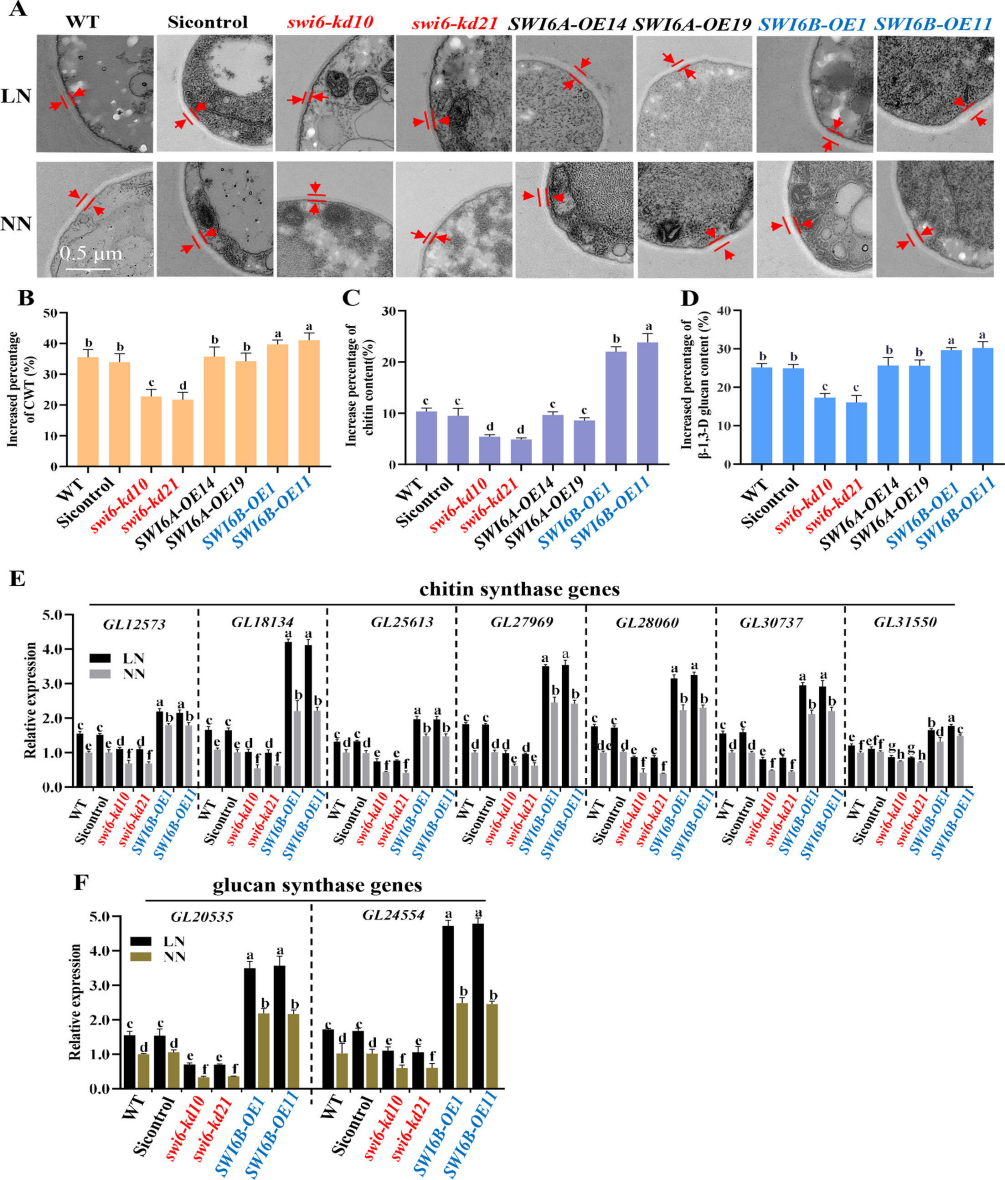
Swi6B upregulates the transcription of polysaccharide synthase genes and promotes the increase of CWT and polysaccharide content under low-nitrogen conditions. (A) The representative CWT pictures of WT and Swi6 relative strains. (B) The increased percentage of cell wall thickness of different genotype strains under low nitrogen relative to that of normal nitrogen. (C, D) The increased percentage of chitin (C) and glucan (D) content of different genotype strains under low nitrogen relative to that of normal nitrogen. (E, F) The relative transcription of the genes coding for chitin synthases (E) and glucan synthases (F). For (A–F), LN, low nitrogen; NN, normal nitrogen. All the data were obtained from WT or Swi6 relative strains grown under LN or NN conditions as indicated. The growth conditions of all strains and mycelia used in the experiments were similar to Fig. 1. For (B–F), values are shown as the mean ± SD (*n* = 3); the statistical significance is represented by different letters corresponding to *P* < 0.05 based on Tukey’s test. *swi6-kd10/21: SWI6* knockdown strains; *SWI6A-OE14/19: SWI6A* overexpression strains; *SWI6B-OE1/11: SWI6B* overexpression strains.

To investigate the role of Swi6B in cell wall remodeling under low nitrogen, the *SWI6B-*overexpressing strains (*SWI6B-OEs*) and Swi6-knockdown strains (*swi6-kds*) were generated. The mycelial cell wall structure of the WT, *SWI6B-OE,* and *swi6-kd* strains under low-nitrogen or normal nitrogen conditions was observed with TEM. Compared with that under normal nitrogen conditions, the cell wall thickness of all the strains increased under nitrogen deficiency conditions (Fig. 2A; Fig. S1A). However, the increase rates of *SWI6B-OE* (39%–41%) were obviously greater than those of the WT (34%–35%), and the increase rates of *swi6-kds* (21%–22%) were significantly lower than those of the WT (Fig. 2B). Notably, the cell wall thickness of *swi6-kds* after low nitrogen treatment (~36–40 nm) was similar to that of WT without low nitrogen treatment (~41 nm), whereas the cell wall thickness of WT after low nitrogen treatment was similar to that of *SWI6B-OEs* before treatment (Fig. S1A). Consistent with the increased cell wall thickness, the *SWI6B-OEs* also presented dramatically increased contents of chitin and β-1,3-D-glucan compared with those of the WT, whereas the swi6-kds knockdown strains presented the lowest increase rates (Fig. 2C and D; Fig. S1B and C). These results demonstrated that Swi6B increases both the cell wall thickness and polysaccharide content in response to low nitrogen. In contrast, there was no significant difference in cell wall thickness or the contents of chitin and β-1,3-D-glucan between *SWI6A-OEs* and WT, indicating that Swi6A does not affect cell wall synthesis under low-nitrogen conditions (Fig. 2C and D; Fig. S1B and C).

To further understand the changes in the expression of chitin and β−1,3-D-glucan in various strains, the transcription of the genes related to chitin and glucan biosynthesis was evaluated by RT-qPCR. Under sufficient nitrogen conditions, the relative transcript levels of eight chitin and two glucan biosynthesis genes were the highest in the *SWI6B-OEs* and lowest in the *swi6-kds* (Fig. 2E and F). Like the contents of chitin and β-1,3-D-glucan, nitrogen deficiency induced the expression of all these genes, but the rates of increase in *SWI6B-OEs* were obviously greater than those in the WT, and the smallest increase was observed in *swi6-kds* (Fig. 2E and F). These results indicate that Swi6B increases cell wall thickness and polysaccharide abundance by regulating the expression of chitin and glucan biosynthesis-related genes and that nitrogen deficiency conditions promote the function of Swi6B.

### GCN4 directly binds to the promoter of *SWI6* to increase *SWI6B* transcription

**Fig 3:**
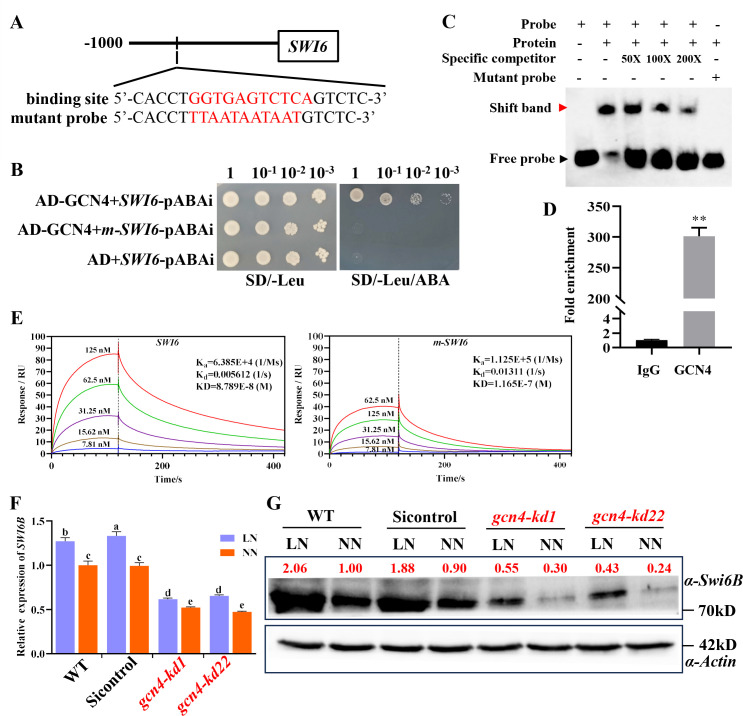
GCN4 directly binds to the promoter region of *SWI6* to promote its transcript and protein accumulation. (A) The diagram of GCN4 binding elements in the *SWI6* promoter. The red characters indicate the binding sites predicted based on Jaspar and used as probes in Y1H and EMSA. The binding site “GGTGAGTTTCCA” was mutated to the sequence “TTAATAATAAT” to generate a mutant probe. (B) Y1H verification of GCN4 interacts with the GBE of the *SWI6* promoter. The promoter fragments were generated by three tandem repeats of the binding site (*SWI6*) or its mutated sequence (*m-SWI6*). (C) EMSA verified the specific binding of GCN4 protein to the binding site of the *SWI6* promoter. Both the probe and mutant probe were biotin-labeled, whereas the cold probe was not biotin-labeled. The number of cold probes is 50, 100, and 200 times that of the labeled probe. (D) ChIP-qPCR confirmed the interaction between GCN4 and the *SWI6* promoter. Immunoprecipitation (IP) was performed using an antibody against GCN4, and the enriched DNA fragments were used as a template for qPCR. Values are shown as the mean ± SD (*n* = 3) of the cycle threshold, and the statistical significance is conducted with Student’s *t* test. (*, *P*  <  0.05; **, *P*  <  0.01). (E) Kinetics detection of binding force between GCN4 protein and *SWI6* promoter. The mutant sequence is the same as with YIH assay. K_a_ and K_d_ represent kinetics data; KD represents affinity. The smaller the KD value is, the stronger the binding ability. “Response” reflects the signal changes on the sensor surface due to molecular interaction. “RU” stands for “response unit” (F) RT-qPCR analysis of the relative expression of *SWI6B* in the WT and GCN4-knockdown (*gcn4-kd1/22*) strains under LN or NN conditions. Values are shown as the mean ± SD (*n* = 3); the statistical significance is represented by different letters corresponding to *P* < 0.05 based on Tukey’s test. (G) Western blotting analysis of the abundance of Swi6B protein in the WT and *gcn4-kd1/22* under LN or NN conditions. The red number indicates the band intensity, and the protein level of WT under NN conditions was set as 1. LN, low nitrogen; NN, normal nitrogen.

Although we previously reported that low nitrogen increases cell wall thickness by increasing the transcription and protein levels of Swi6B, the underlying mechanism is unclear. To address this issue, Y1H library screening was performed to identify the transcription factor that binds with the promoter of *SWI6*. GCN4, a previously reported transcription factor that is activated by low-nitrogen conditions, was identified (Table S2). In the promoter of *SWI6*, a cis-element, “GGTGAGTTTCCA” (designed as a GCN4 binding element, GBE), was predicted as the binding site of GCN4 by Jaspar (Fig. 3A). Different experiments were subsequently conducted to further verify the interaction between GCN4 and GBE. In the Y1H assay, GCN4 interacted with the natural but not mutated promoter of *SWI6* (Fig. 3B). The EMSA revealed that the GCN4 protein directly binds to the GBE probes, whereas the interaction was abolished when the probes carried mutations in GBE (*m-SWI6*) (Fig. 3C). To further verify the binding of GCN4 to the *SWI6* promoter *in vivo*, ChIP-qPCR was performed with a GCN4 antibody and primers covering GBE in the WT strains. The results revealed that GCN4 was highly enriched in the promoter region of *SWI6* (Fig. 3D). The direct and specific interaction between GCN4 and GBE was further confirmed by SPR. The affinity KD value between GCN4 and GBE was 8.789E-8, whereas the KD value between GCN4 and the mutated *m-SWI6* promoter was only 1.165E-7 (Fig. 3E). Furthermore, in the GCN4-knockdown strains, the transcript level of *SWI6B* was significantly reduced, and the rate of *SWI6B* upregulation induced by low-nitrogen conditions was also reduced (Fig. 3F). Consistent with the reduced transcription, the abundance of the Swi6B protein was also dramatically reduced in the two *gcn4-kd* strains (Fig. 3G), whereas there was no significant difference in the transcription or protein level of Swi6A (Fig. S2). These data demonstrated that GCN4 specifically binds to the promoter of *SWI6* and promotes the transcription of *SWI6B*, resulting in increased Swi6B protein.

### Knockdown of GCN4 reduces the increase in cell wall thickness and polysaccharide content under low-nitrogen conditions

**Fig 4:**
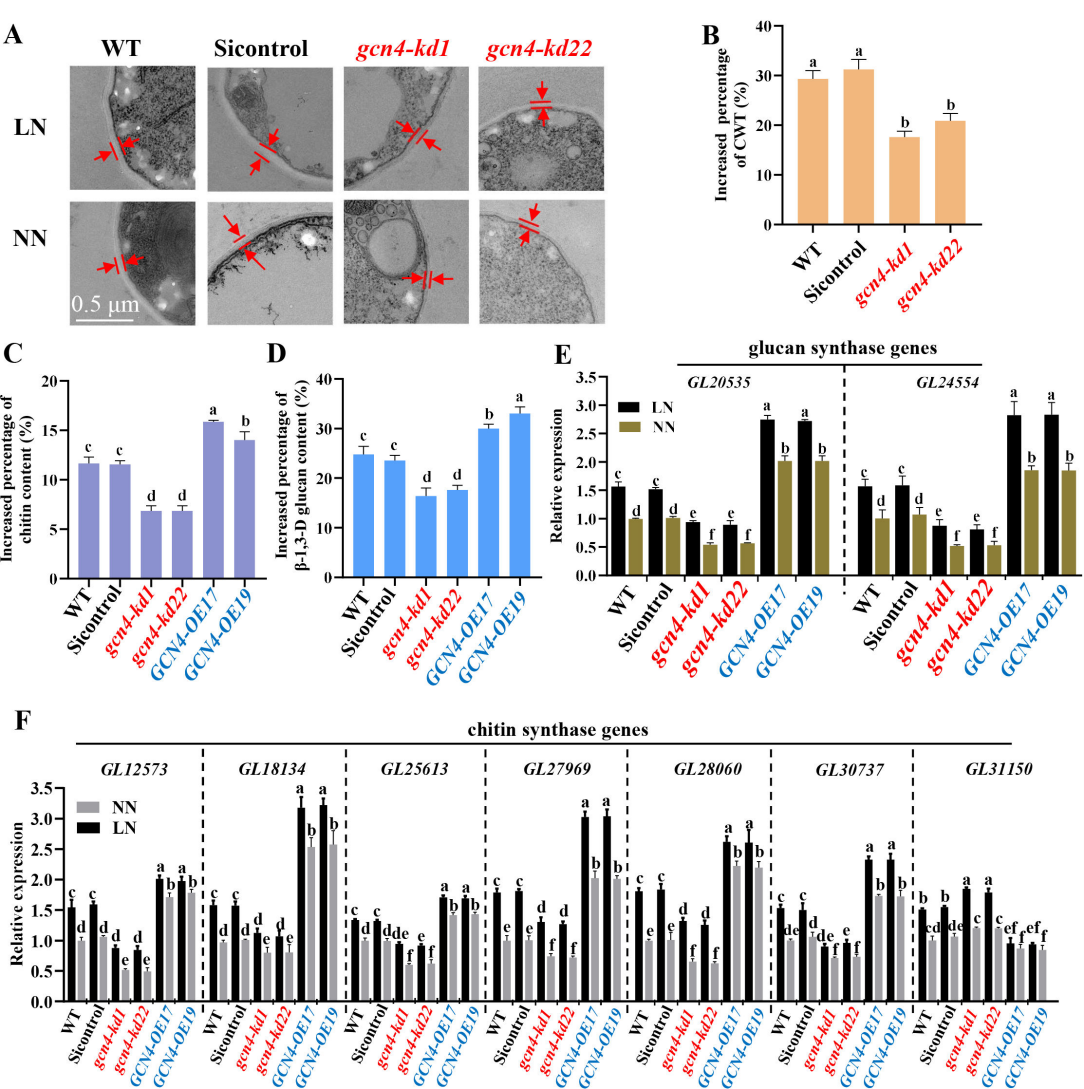
GCN4 increases the CWT and polysaccharide content under low-nitrogen conditions by upregulating the transcription of polysaccharide synthase genes. (A) The representative CWT pictures of WT and GCN4 relative strains under LN or NN conditions. (B) The increased percentage of cell wall thickness of WT and GCN4 relative strains under low nitrogen relative to that of normal nitrogen. (C, D) The increased percentage of chitin (C) and glucan (D) content of indicated genotype strains under low nitrogen relative to that of normal nitrogen. (E, F) RT-qPCR analysis of the relative transcription of genes coding for glucan synthases (E) and chitin synthases (F) in the WT, GCN4 knockdown, and overexpression strains. For (A–F), LN, low nitrogen; NN, normal nitrogen. All the data were obtained from WT or GCN4 relative strains grown under LN or NN conditions as indicated. For (B–F), values are shown as the mean ± SD (*n* = 3); the statistical significance is represented by different letters corresponding to *P* < 0.05 based on Tukey’s test. *gcn4-kd1/22*: GCN4 knockdown strains; *GCN4-OE17/19*: GCN4 overexpression strains.

Owing to the function of GCN4 in promoting the expression of *SWI6B*, we explored the effect of GCN4 on cell wall remodeling with or without nitrogen deficiency. The results revealed that, regardless of the nitrogen concentration, the cell wall thickness of the GCN4-knockdown strain (*gcn4-kds*) was smaller than that of WT and vector control strains (Fig. 4A; Fig. S3A). To further understand the function of GCN4, *GCN4-*overexpressing strains (*GCN4-OEs*) were generated (Fig. S3B and C). Compared with WT, *GCN4* knockdown reduced the concentrations of chitin and β-1,3-D-glucan, whereas *GCN4* overexpression increased their contents (Fig. S3D and E). In addition, similar to the results of Swi6B, the percentages of increased cell wall thickness and polysaccharide in WT were significantly greater than those in *gcn4-kds* but dramatically lower than those in *GCN4-OEs* (Fig. 4C and D). Compared with those in the WT strain, the transcript levels of chitin and glucan biosynthesis genes were downregulated in *gcn4-kd* strains but upregulated in *GCN4-OEs*, which exhibited similar expression patterns to those of Swi6B-related strains (Fig. 4E and F). On the basis of these results, we concluded that GCN4 regulates the process of cell wall remodeling under nitrogen deficiency conditions by directly promoting the expression of *SWI6B* and thus increasing the function of Swi6B.

## DISCUSSION

**Fig 5:**
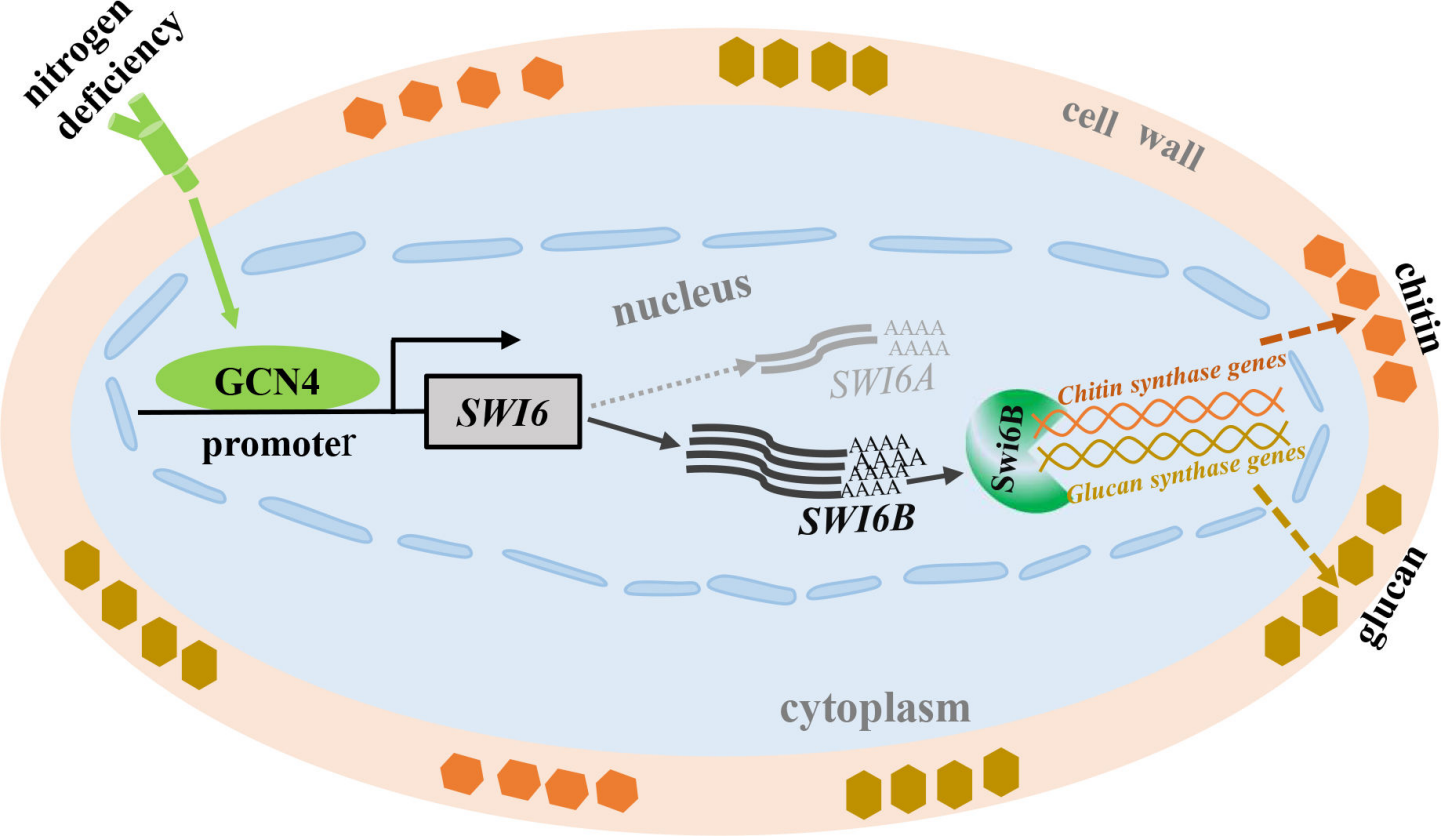
Schematic diagram of the GCN4-Swi6B module regulates cell wall remodeling under nitrogen deficiency environments. In optimal environments, GCN4 binds to the promoter region of the *SWI6* and promotes the transcription of *SWI6B*. When the environments became nitrogen deficient, low nitrogen activates the transcription of GCN4 to further promote the expression and thus protein abundance of Swi6B. The increased Swi6B will upregulate the transcription of the chitin and glucan synthase genes, resulting in the accumulation of polysaccharide and thus an increase in cell wall thickness.

Cell wall synthesis plays a crucial role in the response to environmental stress. The composition of the cell wall is highly regulated depending on the environmental conditions. The complicated network of signaling enables the cell wall integrity pathway to be activated by numerous types of stresses and provides the necessary responses to maintain fungal cell viability (17). As a result, the cell wall thickness and polysaccharide content are regulated in response to environmental stress. Nitrogen and iron deficiency increases cell wall thickness in some algae. The *Candida albicans* cells grown under iron-depleted conditions have thicker cell walls, whereas those grown on lactate have thinner cell walls (43). Previously, we have reported that the transcription of *GCN4* is induced by nitrogen deficiency conditions (31). However, the relationship between GCN4 and cell wall remodeling is unclear. In this study, we found that the cell wall of *G. lucidum* under low-nitrogen conditions was thicker than that under normal-nitrogen conditions (Fig. 1A and B). Furthermore, similar to plants and microalgae (23, 44), nitrogen-limited conditions also increased the polysaccharide content of the cell wall in *G. lucidum* (Fig. 1C and D). On the basis of our results, we propose a model for how the GCN4-Swi6B module regulates cell wall remodeling under nitrogen deficiency conditions (Fig. 5). In this model, nitrogen deficiency increases both the transcription and protein levels of GCN4. The increased GCN4 bound to the promoter region and improved the transcription of *SWI6B*, leading to an increased level of the Swi6B protein (Fig. 3). Finally, the Swi6B protein activates the expression of several chitin and glucan synthesis genes, resulting in increased contents of chitin and β−1,3-D-glucan (Fig. 2), ultimately leading to a thicker cell wall (Fig. 1).

Research on the regulation of cell wall synthesis mechanisms in fungi has focused mainly on the cell wall integrity pathway, which is mediated by the mitogen-activated protein kinase (MAPK) cascade. Different cell wall injury conditions in *S. cerevisiae* trigger rescue mechanisms related to transcriptional reprogramming through the cell wall integrity pathway (45, 46). As a key transcription factor of the cell wall integrity pathway, Swi6 has been reported to be phosphorylated by PKC (21) and to form trimeric complexes with the MAPKs SLT2 and Swi4 (47–49), both of which result in the activation of its transcriptional function. In *G. lucidum*, Swi6B is predominantly localized in the nucleus and can promote the synthesis of chitin and β-1,3-D-glucan in the cell wall, whereas Swi6A is mainly localized in the cytoplasm and has no effect on cell wall synthesis (22), indicating that alternative splicing of *SWI6* also plays an important role in maintaining cell wall integrity. Although GCN4 directly binds and activates the promoter of *SWI6*, only the transcript of *SWI6B* was significantly upregulated under nitrogen deficiency conditions (Fig. 3F and 2A). These data demonstrated that *G. lucidum* takes advantage of transcriptional regulation and alternative splicing to synergistically activate *SWI6B*, ensuring survival in a nitrogen deficiency environment. As shown in Fig. 3G, the level of the Swi6B protein was almost undetectable in *gcn4-kd* strains under adequate nitrogen conditions, indicating that GCN4 can promote the expression of Swi6B. After the nitrogen deficiency, the expression of Swi6B in *gcn4-kd* strains increased, but it did not recover to the WT level, indicating that the increased expression of Swi6B under nitrogen deficiency conditions is partially dependent on GCN4. Our results provide another regulatory mechanism of *SWI6* and confirm that GCN4 is an important component of the cell wall integrity pathway.

Cell walls are dynamic structures whose composition and integrity can undergo cell wall remodeling in response to environmental challenges and developmental cues (50). The bacterial pathogen *Staphylococcus aureus* responds to the host environment by synthesizing a thick peptidoglycan cell wall, which protects the bacterium from membrane-targeting antimicrobials and the immune response (51). When exposed to high-salt conditions, *Aspergillus sydowii* enhances chitin glucan synthesis, resulting in the formation of thick, hard, and hydrophobic cell walls. This structural rearrangement reduces permeability, enabling the fungus to adapt to conditions of high salinity and salt deficiency, providing a robust mechanism to withstand external pressures (52). During the growth of *G. lucidum*, it encounters more than one type of abiotic stress. Many studies have reported that the resistance tolerance of organisms is increased under nitrogen-limiting conditions (53–58). The increased cell wall thickness may be one of the mechanisms by which organisms increase their ability to resist stress. It is possible that low-nitrogen conditions function as a preliminary signal of adverse environments in the natural habitats, triggering the additional synthesis of the cell wall to prepare for the upcoming harsh environments. Therefore, the GCN4-Swi6 regulatory network may play an adverse role in resistance by regulating the cell wall integrity pathway when fungi respond to nitrogen deficiency stress. Our results provide valuable insights into how fungal cell walls respond to environmental cues through signal transduction pathways, contributing to a deeper understanding of fungal cell wall modulation mechanisms.

## Data Availability

The authors confirm that the data supporting the findings of this study are available within the article and its supplemental material.

## SUPPLEMENTAL MATERIAL

Fig. A1 aem.00164-25-s0001.tif Swi6B upregulates the cell wall thickness (CWT) and polysaccharide content under low nitrogen conditions.

Fig. A2 aem.00164-25-s0002.tif GCN4 does not promote the transcription and protein level of Swi6A.

Fig. A3 aem.00164-25-s0003.tif GCN4 increases the cell wall thickness and polysaccharide content under low nitrogen.

Tables S1 and S2 aem.00164-25-s0004.docx Primers used for gene expression analysis; transcription factors selected by yeast one-hybrid library screening.

Supplemental legends aem.00164-25-s0005.docx Legends for Fig A1 to A3.

ASM does not own the copyrights to Supplemental Material that may be linked to, or accessed through, an article. The authors have granted ASM a non-exclusive, world-wide license to publish the Supplemental Material files. Please contact the corresponding author directly for reuse.
