# Supplementary figures and images for: The GCN4-Swi6B module mediates low nitrogen-induced cell wall remodeling in Ganoderma lucidum
Source: Appl Environ Microbiol. 2025 Mar 27;91(4):e00164-25. doi: 10.1128/aem.00164-25 (PMC12016525; doi:10.1128/aem.00164-25)

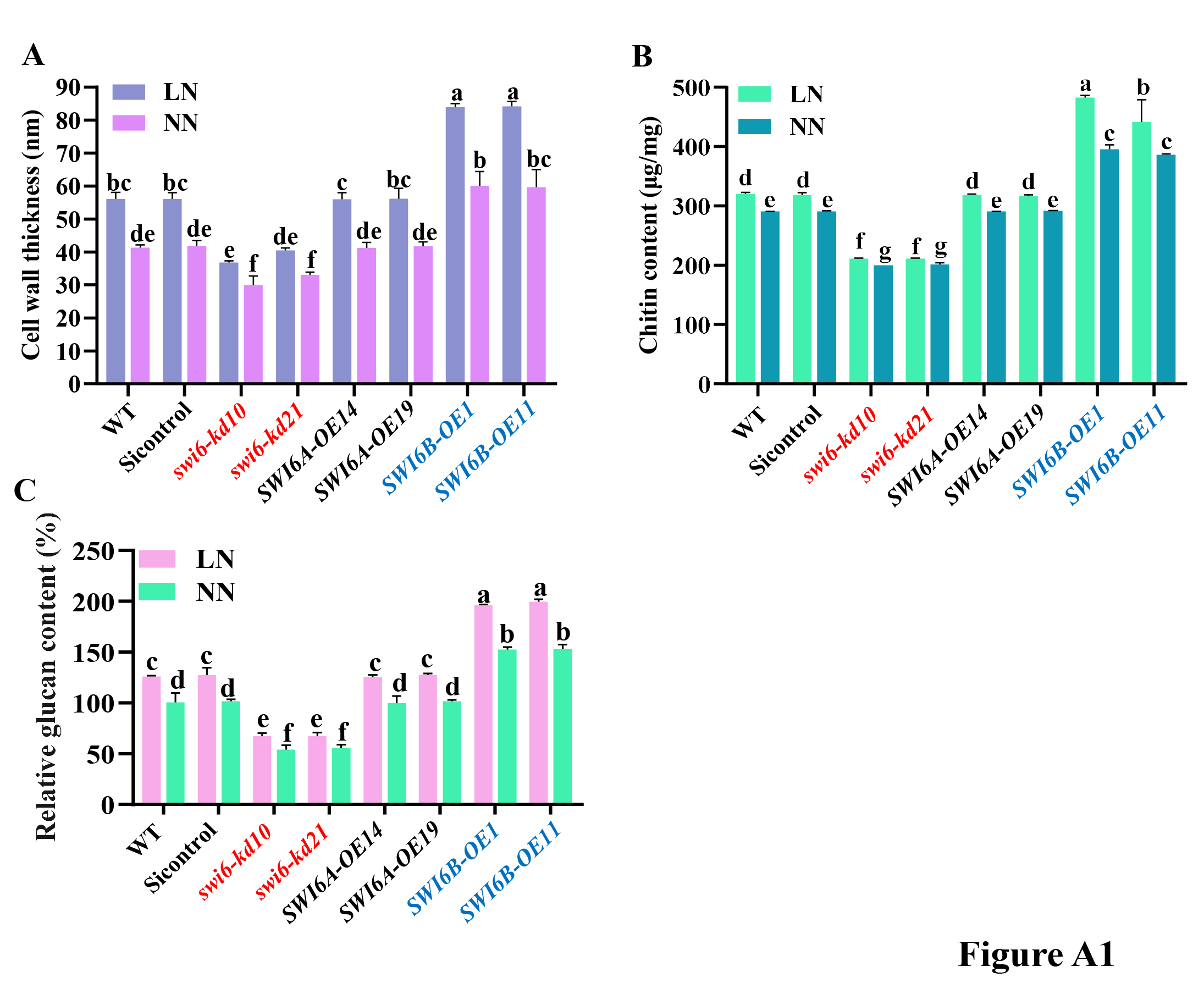

Supplement: Fig. A1 — Swi6B upregulates the cell wall thickness (CWT) and polysaccharide content under low nitrogen conditions. [file aem.00164-25-s0001.tif]

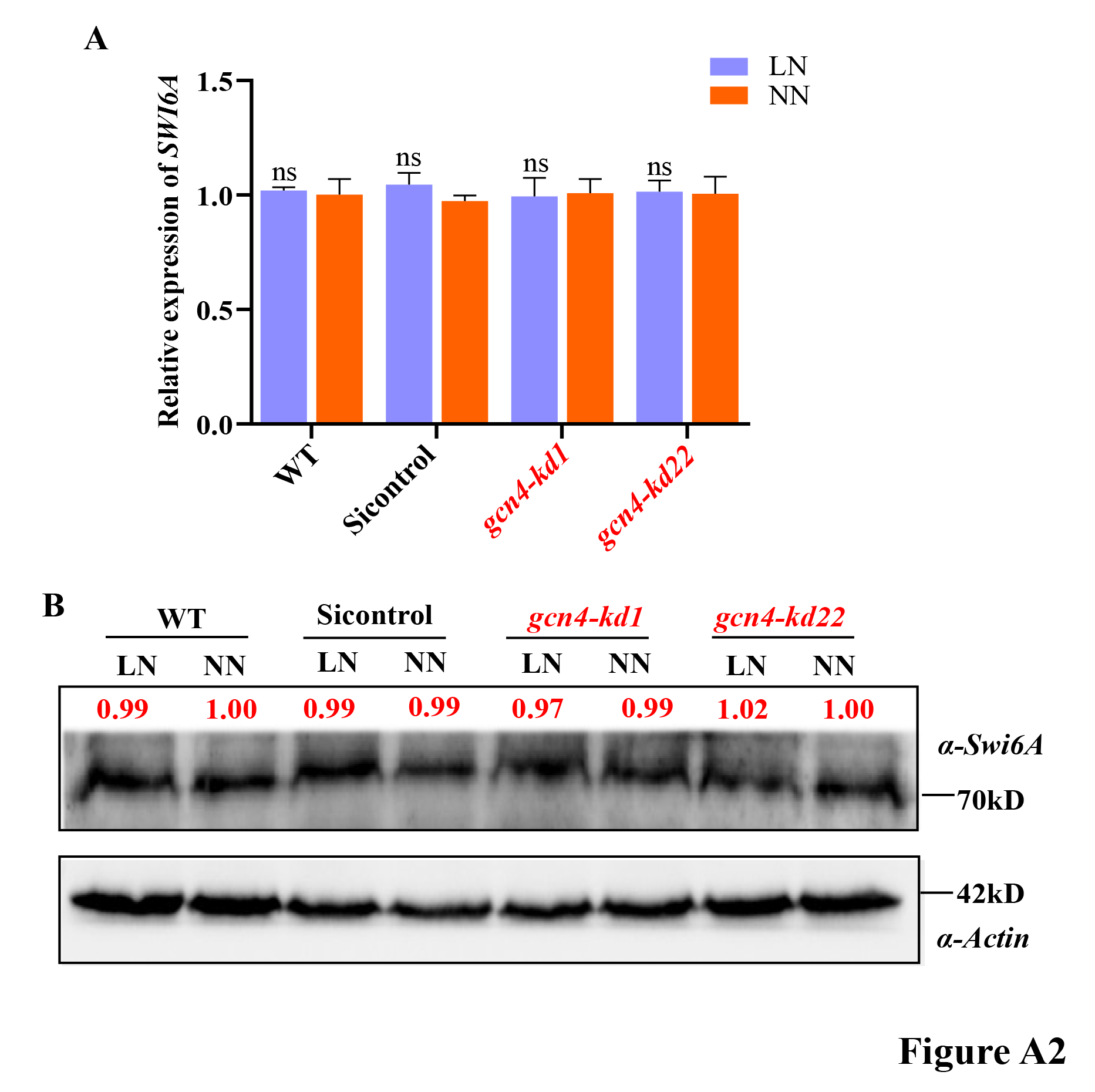

Supplement: Fig. A2 — GCN4 does not promote the transcription and protein level of Swi6A. [file aem.00164-25-s0002.tif]

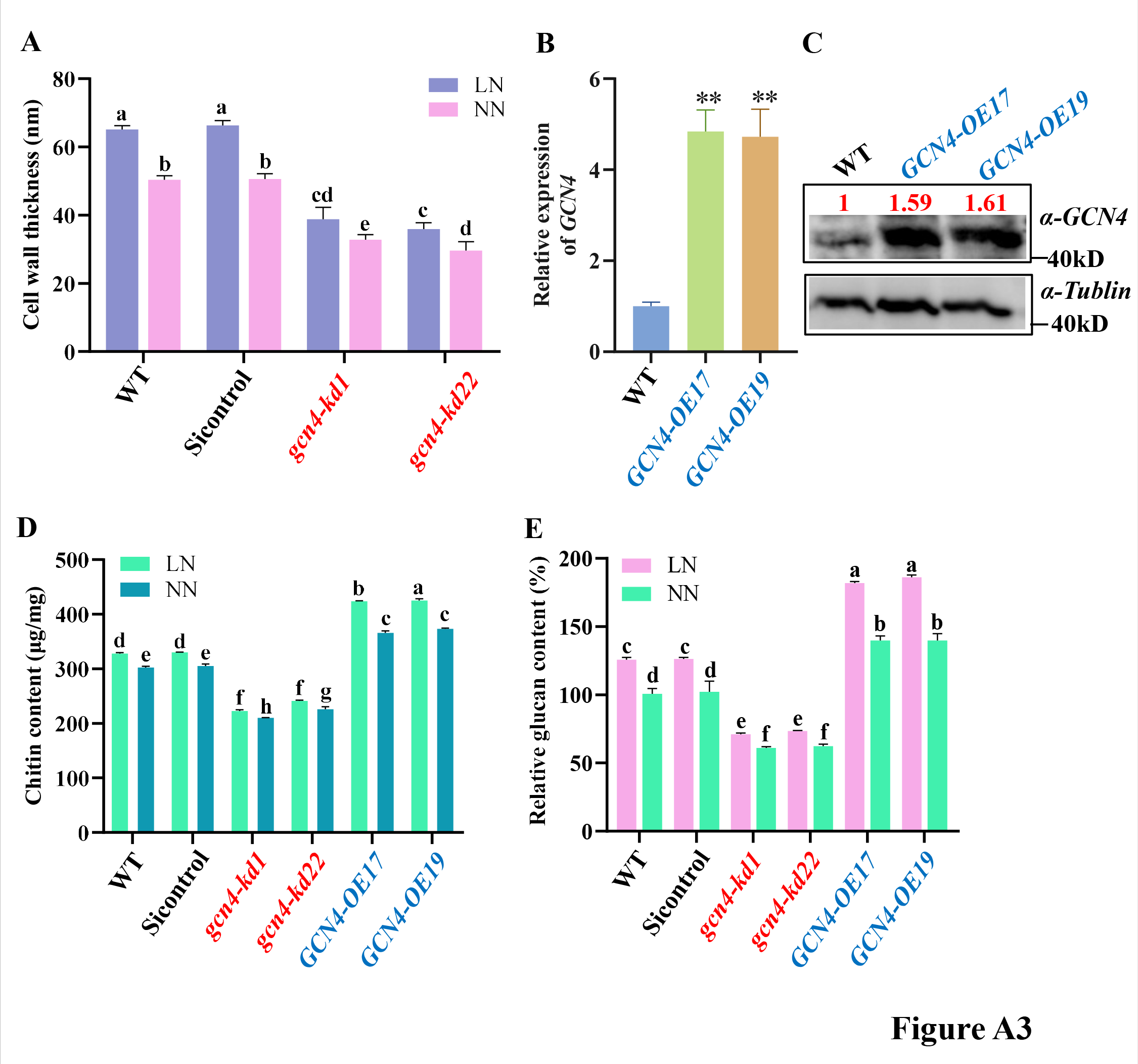

Supplement: Fig. A3 — GCN4 increases the cell wall thickness and polysaccharide content under low nitrogen. [file aem.00164-25-s0003.tif]
