# Supplementary material for: The GCN4-Swi6B module mediates low nitrogen-induced cell wall remodeling in Ganoderma lucidum
Source: Appl Environ Microbiol. 2025 Mar 27;91(4):e00164-25. doi: 10.1128/aem.00164-25 (PMC12016525; doi:10.1128/aem.00164-25)
Supplement: Tables S1 and S2 — Primers used for gene expression analysis; transcription factors selected by yeast one-hybrid library screening. [file aem.00164-25-s0004.docx]

**Table A1.**  Primers used for gene expression analysis

| Primer | Sequence (5’ to 3’) | Description |
| --- | --- | --- |
| RT-*18S*-F | TATCGAGTTCTGACTGGGTTGT | Detects the *18S* expression |
| RT-*18S*-R | ATCCGTTGCTGAAAGTTGTAT |  |
| RT-*GL15273*-F | CTTCATTTCCCGCATTATCTG | Detects the putative chitin synthase expression |
| RT-*GL15273*-R | CGACTGTGCCACCGTAGTT |  |
| RT-*GL18134*-F | CTCGTGCCTGTCCAGATTAT | Detects the putative chitin synthase expression |
| RT-*GL18134*-R | AAGGTGCGGGTCGTTGTAG |  |
| RT-*GL25613*-F | TTGCTGTCCTGGGTCTTCC | Detects the putative chitin synthase expression |
| RT-*GL25613*-R | GTGAGCTTCGCCCTTACGT |  |
| RT-*GL27969*-F | AACCAAGCCGCCGAAACAG | Detects the putative chitin synthase expression |
| RT-*GL27969*-R | CGAACCAATGAACCGGACAA |  |
| RT-*GL28060*-F | CCTCTTCGCAATGGGCAACA | Detects the putative chitin synthase expression |
| RT-*GL28060*-R | TTGGCAGCCTGGATGGAAC |  |
| RT-*GL30737*-F | TATTCGTCCGTACTATGAACTCC | Detects the putative chitin synthase expression |
| RT-*GL30737*-R | CGATACCCTCCTGATAGCAAC |  |
| RT-*GL31550*-F | CAACCGTGACGCCTGACTC | Detects the putative chitin synthase expression |
| RT-*GL31550*-R | AACTCCAAAGGGTTTCCATAA |  |
| RT-*GL20535*-F | GCTGCTGTATGTGACGCTGAC | Detects the putative glucan synthase expression |
| RT-*GL20535*-R | TGGTGCGGGTTGAATAAGAA |  |
| RT-*GL24554*-F | GTTCGTAAAGGGAAAGGT | Detects the putative glucan synthase expression |
| RT-*GL24554*-R | AGCAGGATGTAAAGCCAG |  |
| RT-*SWI6B*-F | CCCCATCTACATCCACAGCTG | Detects the *SWI6B* expression |
| RT- *SWI6B*-R | CGCGATCGGTCCAGCATT |  |
| RT-*SWI6A*-F | TGTTTCACCTGGCCCACA | Detects the *SWI6A* expression |
| RT-*SWI6*-R | CGCGATCGGTCCAGCATT |  |
| *pSWI6*-pABAi-F | GATTTTGGAGAAGGAGAT | *SWI6* promoter fragment for YIH validation. |
| *pSWI6*-pABAi-R | CAGATTATGGTGGGATGC |  |
| *pSwi6*-Biotin-F | GAAAGTACCAGGGCACCTGGTGAGTCTCAGTCTCGGTGCCATG | Biotin-labeled *SWI6* promoter fragment for EMSA validation. |
| *pSwi6*-Biotin-R | CATGGCACCGAGACTGAGACTCACCAGGTGCCCTGGTACTTTC |  |
| *pSwi6*-M-Biotin-F | GAAAGTACCAGGGATAAATTTATTATATAGTCTCGGTGCCATG | Biotin-labeled *SWI6* promoter mutant fragment for EMSA validation. |
| *pSwi6*-M-Biotin-R | CATGGCACCGAGACTATATAATAAATTTATCCCTGGTACTTTC |  |
| *pSwi6*-F | GAAAGTACCAGGGCACCTGGTGAGTCTCAGTCTCGGTGCCATG | Competing fragment of *SWI6* promoter for EMSA validation. |
| *pSwi6*-R | CATGGCACCGAGACTGAGACTCACCAGGTGCCCTGGTACTTTC |  |
| RT-*GCN4*-F | CACTTGATCCTACGTCCCTTTAT | Detects the *GCN4* expression |
| RT-*GCN4*-R | GGTTCTTCCGTGTACCAGTT |  |
| *GCN4*-OE-F | CGGGATCCATGCTCTCGCAAAACCCTCAG | Get the full length of  *GCN4* gene |
| *GCN4*-OE-R | GCTCTAGATCATGTTGTATCGAACGAC |  |
| *GCN4*-kd-F | TCGGGTACCTGCACAACTTAACGGAGGAG | Get the knockdown  fragment of *GCN4* gene |
| *GCN4*-kd-R | GCTAACTAGTGCATAAAGGGACGTAGGAT |  |

**Table A2. Transcription factors selected by yeast one-hybrid library screening.**

| **Genes** | **Descriptions** |
| --- | --- |
| *Gl15296* | ACE1 transcription factor |
| *Gl15449* | STE like transcription factor |
| *Gl17608* | Fungal Zip1-like transcription factors |
| *Gl25443* | CAT8 transcription factor |
| *Gl20629* | GAL4-like Zn(Ⅱ)2Cys6 transcription factor domain-containing protein |
| *Gl22824* | TATA box binding protein (TBP) |
| *Gl23501* | Fungal specific transcription factor domain |
| *Gl28816* | C2H2 Zinc-finger transcription factor |
| *Gl28195* | GCN4 transcription factor |
| *Gl26798* | MBP1 transcription factor |
| *Gl25464* | CRZ1 transcription factor |
| *Gl24529* | SKN7 transcription factor |
| *Gl19424* | CreA transcription factor |
