# Supplementary material for: The GCN4-Swi6B module mediates low nitrogen-induced cell wall remodeling in Ganoderma lucidum
Source: Appl Environ Microbiol. 2025 Mar 27;91(4):e00164-25. doi: 10.1128/aem.00164-25 (PMC12016525; doi:10.1128/aem.00164-25)
Supplement: Supplemental legends — Legends for Fig A1 to A3. [file aem.00164-25-s0005.docx]

**Supplemental legends**

**Figure A1.** Swi6B upregulates the cell wall thickness (CWT) and polysaccharides content under low nitrogen condition. **(A)** Values of CWT of WT and Swi6 relative strains under LN or NN conditions. Values are shown as means ± SD (n = 30). **(B-C)** The content of chitin (B) and glucan (C) of WT and Swi6 relative strains under LN or NN conditions. Values ​​are shown as the mean ± SD (n = 3). The statistical significance is represented by different letters corresponding to P <0.05 based on Tukey’s test. **Related to Figure 2**.

**Figure A2.** GCN4 does not promote the transcription and protein level of Swi6A. **(A)** qRT-PCR analysis the relative expression of *SWI6B* in WT and GCN4 knockdown (*gcn4-kd1/22*) strains under LN or NN conditions. Values ​​are shown as the mean ± SD (n=3), the statistical significance is represented by different letters corresponding to P <0.05 based on Tukey’s test. **(G)** Western blotting analysis of the abundance of Swi6A protein in WT and *gcn4-kd1/22* under LN or NN conditions. The red data indicate the band intensity, and the protein level of WT under NN condition was set as 1. **Related to Figure 3.**

**Figure A3.** GCN4 increase the cell wall thickness and polysaccharides content under low nitrogen. **(A)** Values of cell wall thickness of WT and GCN4 relative strains under LN or NN conditions. Values are shown as means ± SD (n = 30). **(B)** qRT-PCR analyzes the transcription level of *GCN4* in WT and GCN4 overexpression strains. Values are shown as the mean ± SD (n = 3), the statistical significance is conducted with Student's t test. (*, P < 0.05; **, P < 0.01). **(C)** Western blotting analyzes the protein level of GCN4 in WT and GCN4 overexpression strains. **(D-E)** The content of chitin (D) and glucan (E) of WT and GCN4 relative strains under LN or NN conditions. Values ​​are shown as the mean ± SD (n = 3). The statistical significance is represented by different letters corresponding to P <0.05 based on Tukey’s test. **Related to Figure 4**.
